# Outcomes of Gonioscopy-Assisted Transluminal Trabeculotomy (GATT) in Advanced Glaucoma: A Retrospective Analysis

**DOI:** 10.3390/medicina61030444

**Published:** 2025-03-03

**Authors:** Gülizar Soyugelen, Umay Güvenç, Ayşe Burcu

**Affiliations:** Affiliation: Ankara Training and Research Hospital, Ophthalmology Clinic,06340 Ankara, Türkiye; umay.guvenc@gmail.com

**Keywords:** advanced glaucoma, gonioscopy-assisted transluminal trabeculotomy (GATT), pseudoexfoliative glaucoma (PXG), peripapillary vessel density (VD)

## Abstract

*Background and Objectives:* The search for less invasive and more effective methods in the surgical treatment of glaucoma continues. For advanced glaucoma, all surgical options carry a high risk of complications and vision loss. The aim of this study was to evaluate the outcomes of gonioscopy-assisted transluminal trabeculotomy (GATT) surgery in advanced glaucoma. *Materials and Methods*: In this retrospective study, advanced open-angle glaucoma (OAG) patients were followed up on the 1st day, 1st week, and 1st month, then monthly for 6 months and then every 3 months after GATT surgery. Outcomes at 6 and 24 months were analyzed to evaluate early and long-term surgical success. Surgical success was defined as a ≥20% intraocular pressure (IOP) reduction from baseline, final IOP ≤21 mmHg, and no need for additional glaucoma surgery. Pre- and postoperative measurements included IOP, retinal nerve fiber layer (RNFL) thickness by optical coherence tomography (OCT), peripapillary vessel density (VD) via optical coherence tomography angiography, and visual field (VF) tests. *Results:* Among 44 advanced glaucoma patients (61.4% pseudoexfoliative glaucoma), surgical success was 81.8% at 6 months and 76.5% at 24 months. Mean IOP decreased from 26.9 ± 10.4 mmHg preoperatively to 13.8 ± 8.3 mmHg at 3 months (40.36% reduction, *p* < 0.001) and 13.9 ± 4.0 mmHg at final follow-up (42.12% reduction, *p* < 0.001). Disease progression was absent in 66% of patients. BCVA initially declined (0.61 ± 0.36 to 0.41 ± 0.33 logMAR at 3 months, *p* = 0.011) but returned to baseline (0.59 ± 0.35 logMAR at final follow-up, *p* = 1.00). Glaucoma medications decreased by 66.2%, and peripapillary VD remained stable (*p* > 0.05). The most common complication was mild hyphema (34.1%), which resolved without intervention; only one patient (2.3%) experienced vision-threatening complications (wipe-out phenomenon in degenerative myopia). *Conclusions*: GATT is a safe and effective alternative to trabeculectomy for advanced glaucoma, achieving significant IOP reduction with stable VD and low rates of serious complications. This study provides novel insights by offering long-term (24-month) follow-up data, evaluating peripapillary VD stability, and specifically assessing GATT outcomes in an advanced glaucoma cohort. However, caution is advised in patients with additional ocular pathologies.

## 1. Introduction

Glaucoma is one of the leading causes of irreversible blindness and is estimated to affect approximately 111.8 million people worldwide by 2040 [1]. Patients may present at very advanced stages because of the asymptomatic pattern and insidious progression. One third of patients diagnosed by screening have advanced glaucoma in at least one eye [2].

In advanced glaucoma, IOP must be lowered aggressively to prevent vision loss. The Advanced Glaucoma Intervention Study (AGIS) shows that maintaining an IOP below 18 mmHg, and even below 14 mmHg if possible, is associated with reduced disease progression [3]. National Institute for Health and Care Excellence (NICE) guidelines recommend immediate surgical intervention in advanced glaucoma because of the increased cost and risk of blindness [4,5].

Although trabeculectomy, the most common glaucoma surgery, provides effective IOP reduction, it is associated with serious complications, such as hypotony, retinal detachment, and endophthalmitis, and these risks are increased in advanced glaucoma [6]. Bleb-independent procedures with a more favorable safety profile are needed to preserve vision in glaucoma patients [7,8]. Gonioscopy-assisted transluminal trabeculotomy (GATT), first described by Grover et al. in 2014, is an ab interno conjunctiva-sparing minimally invasive glaucoma surgery (MIGS) that reduces resistance in the trabecular meshwork and inner wall of Schlemm’s canal [9].

Several studies have reported promising long-term results for GATT. Grover et al. (2018) found that GATT achieved a mean IOP reduction of 37.3% at 24 months, with an overall success rate exceeding 75% in primary open-angle glaucoma (POAG) patients [10]. Rahmatnejad et al. (2017) similarly reported a mean IOP reduction of 36% at one year, with 63% of patients achieving surgical success [11]. While these findings support the efficacy of GATT in mild-to-moderate glaucoma, data on its long-term outcomes in advanced glaucoma remain limited.

Given the progressive nature of advanced glaucoma and the limited availability of data evaluating GATT in this patient population, there remains an urgent need to assess its long-term efficacy and safety [8]. This study aims to address this gap by providing comprehensive clinical outcomes of GATT in patients with advanced open-angle glaucoma, offering valuable insights into its role as a potential alternative to traditional surgical interventions.

## 2. Materials and Methods

This retrospective study was approved by the Ankara Training and Research Hospital’s Institutional Review Board on 20 November 2024 with approval number E-24/302 and was carried out in complete accordance with the Declaration of Helsinki. Patients who underwent gonioscopy-assisted transluminal trabeculotomy (GATT) surgery for advanced open-angle glaucoma were analyzed. This study included patients with open-angle glaucoma whose intraocular pressure (IOP) was uncontrolled despite maximal medical therapy and who showed signs of glaucoma progression on retinal nerve fiber layer (RNFL) and/or visual field tests. We excluded those with chronic angle closure, neovascular glaucoma, undefined trabecular meshwork (TM), corneal decompensation, and unstable intraocular lens and patients requiring anticoagulant therapy. These conditions were omitted as they could interfere with surgical success or postoperative assessments. Chronic angle closure and neovascular glaucoma were excluded due to their compromised trabecular outflow, which limits the effectiveness of GATT. Undefined TM and corneal decompensation hinder intraoperative visualization, increasing the risk of incomplete surgery and inaccurate postoperative imaging. Unstable IOLs may alter aqueous humor dynamics and affect IOP control, confounding surgical outcomes. Finally, systemic anticoagulation increases the risk of persistent hyphema, which can lead to IOP fluctuations and prolonged inflammation, potentially impacting this study’s findings.

### 2.1. Definition of “Advanced Glaucoma”

The International Classification of Diseases (ICD) defines advanced glaucoma as glaucomatous optic disk appearance and visual field loss in both the upper and lower hemispheres or defects in the fixation zone (within 5°) [12].

### 2.2. Ophthalmic Examination

Goldmann applanation tonometry was used for pre- and postoperative IOP measurements in all patients. Anterior and posterior segment findings were evaluated and recorded by biomicroscopic and fundus examination. Visual field analysis was performed with the 24-2C SITA-FAST strategy using ZEISS Humphrey Field Analyzer 3 (Carl Zeiss Meditec AG, Jena, Germany). Retinal nerve fiber layer thickness (RNFL) was measured with Heidelberg Spectralis OCT (Heidelberg Engineering GmbH, Heidelberg, Germany). To identify patients with “floor effect” during follow-up and for more accurate progression assessment, macular and peripapillary vessel density was analyzed with AngioVue RTVue-XR OCT angiography (OCTA) (Optovue Inc., Fremont, CA, USA).

### 2.3. Surgical Procedure

Before surgery, all patients underwent gonioscopy to confirm that the angle structures were visible (open angle). Surgeries were performed and recorded by a single surgeon (GS) at the Ophthalmology Department of Ankara Training and Research Hospital using a standardized protocol. After surgical preparations were completed, the patient’s head was slightly elevated to reduce episcleral venous pressure. Two paracenteses were created in the temporal and superior quadrants with a 23-G microsurgical blade and an ocular viscoelastic device (OVD) was introduced into the anterior chamber. After tilting the patient’s head and microscope and observing the angle structures with a Swan-Jacob goniolens, a 1–2 mm goniotomy was created with a microvitreoretinal (MVR) blade. The blunted tip of the 5-0 prolene suture was advanced circumferentially along Schlemm’s canal (SC). When complete passage was feasible, a 360° trabeculotomy was performed; if there was difficulty in passing the suture, a 180° trabeculotomy was applied instead. The most common intraoperative challenges preventing full 360° cannulation included segmental obstruction of Schlemm’s canal, trabecular meshwork fibrosis, anatomical variations, and poor visualization of the angle. In such cases, 180° trabeculotomy was performed to maximize outflow restoration rather than abandoning the procedure. At the end of surgery, OVD was injected into the anterior chamber to prevent blood reflux from the episcleral veins.

### 2.4. Study Outcomes

The primary outcome was surgical success. The secondary outcomes were change in IOP, change in best corrected visual acuity (BCVA), number of glaucoma medications used, and complications. Statistics were presented based on preoperative, 3rd-month, and final IOP and BCVA. Tertiary outcomes were IOP spike rate, progression, change in VF, and change in OCTA. An IOP spike was defined as an IOP of more than 25 mmHg in the first postoperative month. Progression was defined as a thinning of 5 µm or more in at least two consecutive RNFL measurements or a deterioration in visual field progression analysis. Factors influencing postoperative outcomes and complications were evaluated with correlation analysis.

### 2.5. Surgical Success

Surgical success was defined as achieving a reduction in intraocular pressure (IOP) of more than 20% from preoperative levels or maintaining an IOP of ≤21 mmHg with/or without antiglaucoma medications and no need for further glaucoma surgery.

### 2.6. Statistical Analysis

IBM SPSS Statistics for Windows, Version 22.0 (IBM Corp., Armonk, NY, USA) software was used to analyze the data obtained in this study. Continuous data were expressed as mean ± standard deviation (SD) and categorical data were expressed as frequency and percentage. The chi-square test was used for nominal data in intergroup comparisons. In intergroup comparisons of continuous variables, the Mann–Whitney U test was used because the data did not meet the assumptions of normal distribution. The normality assumption of the data was tested with the Shapiro–Wilk test. Pre- and postoperative changes in continuous data such as IOP, visual acuity (LogMAR), retinal nerve fiber thickness (RNFL), and peripapillary vessel density (ppVD) were analyzed by a repeated-measures ANOVA test. A significance level of *p* < 0.05 was considered statistically significant. Short- and long-term surgical success rates were calculated as the percentage of patients who achieved success in each follow-up period. The statistical significance of the difference between success rates was analyzed by the McNemar test. Spearman’s correlation coefficient was used for the relationships between preoperative and postoperative variables. A multivariate regression analysis was performed to assess factors influencing surgical success. Independent variables included age, preoperative IOP, extent of the GATT procedure (180° vs. 360°), presence of postoperative complications, and preoperative visual field mean deviation (MD). Regression coefficients (β), *p*-values, and confidence intervals were reported to determine the significance and strength of associations. A *p*-value of <0.05 was considered statistically significant. Prior to regression, Spearman’s correlation analysis was conducted to assess collinearity among independent variables. The regression model was evaluated for accuracy, but none of the predictors were statistically significant.

## 3. Results

### 3.1. Patient Demographic and Clinical Data

A total of 44 patients were included in this study, of whom 84.1% were male. Surgery was performed on the right eye in 51.1% of cases. The most common glaucoma type was pseudoexfoliative glaucoma (PXG) (61.4%, *n* = 27), followed by primary open-angle glaucoma (POAG) (20.5%, *n* = 9), juvenile glaucoma (6.8%, *n* = 3), and pigmentary glaucoma (PG) (6.8%, *n* = 3). The GATT procedure was completed for 360 degrees in 79.5% (*n* = 35) patients and for 180 degrees in 20.5% (*n* = 9) of patients. At a median follow-up of two years, 66.0% of patients demonstrated no progression of their disease in RNFL analysis or visual field. Five patients required additional surgeries due to uncontrolled intraocular pressure (IOP), which included Ahmed Glaucoma Valve implantation in two cases, trabeculectomy in one case, and diode laser cyclophotocoagulation in two cases (Table 1).

### 3.2. Primary Outcomes

Early (6th-month) surgical success was 81.2% when it was defined as achieving an IOP ≤ 21 mmHg or a reduction of ≥20% from preoperative levels without requiring additional glaucoma surgery. The late (2nd-year) surgical success rate was 76.51% with the same criteria.

### 3.3. Secondary Outcomes

Visual Acuity (BCVA): A temporary postoperative decline in visual acuity was observed, but patients returned to baseline levels during follow-up. The mean preoperative, postoperative at the 3rd month, and final BCVAs were 0.61 ± 0.36, 0.41 ± 0.33, and 0.59 ± 0.35 (logMAR), respectively (Table 2).

**Figure 1:**
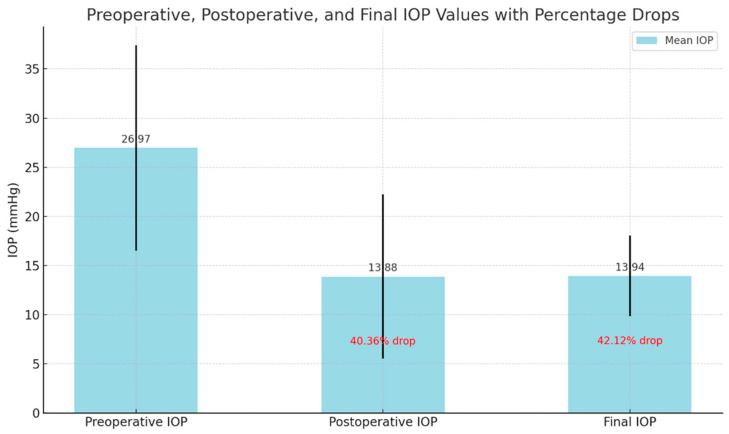
Sustained reduction in intraocular pressure after GATT surgery: This figure shows the mean intraocular pressure (IOP) before surgery, at the 3rd month, and at final follow-up, with significant drops of 40.36% at the 3rd postoperative month and 42.12% at the final follow-up. The percentage drops indicated within the bars demonstrate the effectiveness of the surgery in reducing IOP over time, both in the immediate postoperative period and in the longer-term follow-up.

IOP Reduction: GATT surgery led to a significant and sustained reduction in intraocular pressure (IOP), with a 40.36% decrease at 3 months and 42.12% at the final follow-up (*p* < 0.001). Importantly, postoperative IOP remained consistently below 21 mmHg in the majority of patients, indicating effective pressure control. This level of IOP reduction suggests that GATT may help delay disease progression and reduce the need for additional surgical interventions in patients with advanced glaucoma (Figure 1).

Medication Reduction: The average number of glaucoma medications also decreased from 3.5 preoperatively to 1.18 at the final follow-up, representing a 66.23% reduction in medication use. This reduction suggests that GATT provides adequate IOP control while potentially decreasing the burden of chronic medication use.

Complications: Postoperative complications were observed in 56.8% of patients (25 out of 44). The most common complication, occurring in 34.1% of patients (15 out of 44), was hyphema, which did not require intervention. Two patients (4.5%) required anterior chamber washout due to hyphema. Two patients (4.5%) experienced permanent vision loss, one due to bullous keratopathy and the other to the wipe-out phenomenon. Other complications included Urrets-Zavalia syndrome (2.3%) and hypotonic maculopathy with choroidal folds (2.3%) (Table 1).

### 3.4. Tertiary Outcomes

IOP spike rate: Two patients (4.5%) experienced a postoperative intraocular pressure (IOP) spike, which resolved spontaneously within the first month.

Retinal Nerve Fiber Layer Thickness and Visual Field Parameters: Visual field parameters and retinal nerve fiber layer (RNFL) thickness remained stable, suggesting that the procedure effectively controlled IOP without leading to structural or functional deterioration over time (Table 2).

**Figure 2:**
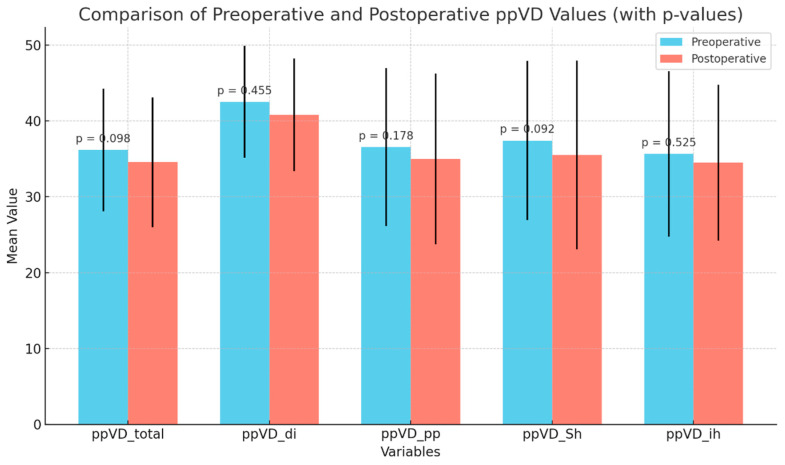
Stability of peripapillary vessel density following GATT surgery: This figure displays the changes in ppVD across various regions before and 2nd year after surgery. The blue bars represent preoperative ppVD values, while the red bars depict postoperative values. Error bars indicate standard deviations for each measurement. Although there was a slight reduction in ppVD following surgery, none of the observed differences were statistically significant (*p* > 0.05), suggesting that the surgery did not result in a significant loss of peripapillary vessel density. This implies that the optic disk vasculature remained stable postoperatively.

OCTA parameters: The data demonstrate a small decrease in vessel density after surgery, but these changes were not significant, suggesting that the surgery did not negatively impact the peripapillary vasculature in a meaningful way (Figure 2).

Progression: Postoperative progression was observed in 34.1% of patients during the follow-up at different intervals. Patients with progression were initially treated with medical treatment, while those without response were treated with additional glaucoma surgery. For patients whose progression detection on OCT was unreliable due to the floor effect, the drop-out in vascular density, which we routinely use, was also considered a determinant for some patients. In the statistical analysis, it was seen that OCTA vascular density did not differ from preoperative values during follow-up. In this regard, it can be said that following surgery, the patients’ courses remained steady.

### 3.5. Factors Influencing Postoperative Outcomes and Complications

Complications and Associated Factors: Patients were divided into two groups based on the presence or absence of postoperative complications to analyze associated variables. No statistically significant relationships were found between complications and factors such as gender, GATT procedure extent, lens status, or preoperative measurements. However, a significant correlation was observed between the presence of complications and postoperative disease progression, with patients experiencing complications having a higher rate of progression (51.9% vs. 15.8%, *p* = 0.029) (Table 3).

Postoperative Progression and Associated Factors: The extent of the GATT procedure was the only statistically significant factor associated with postoperative progression. Patients who underwent a 360-degree GATT procedure had a significantly lower rate of progression compared to those who underwent a 180-degree GATT (*p* = 0.006). Notably, the decision to perform a 180-degree GATT was not always intentional but often due to difficulty in advancing the suture circumferentially along Schlemm’s canal. In total, 34.1% of patients experienced postoperative progression during the follow-up period. Other factors, including gender, lens status, age, and preoperative measurements, were not significantly associated with progression, as no significant differences were observed between progression and no-progression groups (Table 4).

A multivariate regression analysis was performed to determine the impact of various factors on surgical success. However, no statistically significant predictors were identified. The results were as follows:

Age: β = 0.0176, *p* = 0.576;

Preoperative IOP: β = 0.0508, *p* = 0.363;

Extent of GATT (360° vs. 180°): β = 0.9042, *p* = 0.401;

Presence of complications: β = −1.5710, *p* = 0.523;

Preoperative MD: β = −0.0383, *p* = 0.477.

Additionally, there were no significant differences in success rates between patients with primary and secondary glaucoma (*p* = 0.853). The confidence intervals for all variables crossed zero, indicating that none of these factors significantly influenced surgical success.

A comparative analysis of GATT outcomes with previous studies is presented in Table 5. Our study demonstrated a 40.36% reduction in IOP at 3 months and 42.12% at 24 months, which aligns with prior reports on GATT efficacy. Compared to Grover et al. [10], who reported a 37.3% IOP reduction at 24 months, our findings indicate a slightly greater reduction, particularly in an advanced glaucoma cohort. Similarly, Wan et al. [13] observed a 43.7% reduction, which is comparable to our results. When compared to studies focusing on advanced or refractory glaucoma, such as Wang et al. [7] and Dar et al. [8], our results fall within a similar range of 41.1–44.1% IOP reduction at 24 months. Additionally, our surgical success rate of 76.5% at 24 months closely mirrors the 75.3% reported by Grover et al. [10] and the 72.0% in Dar et al. [8]. Furthermore, studies by Aktaş et al. provide additional validation of our findings [14,15].

## 4. Discussion

This study was conducted to evaluate the efficacy and safety of GATT surgery in advanced glaucoma after a mean follow-up of two years. Early and late surgical success rates were found to be 81.2% and 76.5%, respectively. The fact that glaucoma progression was not observed in 66% of the patients during the follow-up period supports the efficacy of the procedure.

### 4.1. Comparison to Other Studies

When compared to previous GATT studies, our findings align with the results from Grover et al. (2018), who reported a 37.3% reduction in IOP over 24 months [10]. Similarly, Wan et al. (2022) observed a 43.7% reduction, which is comparable to our study [13]. Studies focusing on advanced or refractory glaucoma, such as Wang et al. (2023) and Dar et al. (2023), reported 41.1–44.1% IOP reduction, reinforcing the conclusion that GATT provides long-term efficacy in advanced cases [7,8]. In particular, Aktaş et al. evaluated GATT in advanced glaucoma and found a 44.7% IOP reduction at 3 months and 43.2% at 24 months, with a 79.3% success rate, which closely aligns with our findings [15]. Similarly, in a separate study, Aktaş et al. (2019) investigated prolene-assisted GATT in moderate-to-advanced glaucoma and reported nearly identical success rates, further supporting the effectiveness of the procedure across different surgical approaches [14]. 

GATT has been shown to be an effective method of reducing IOP and the number of glaucoma medications in patients with open-angle glaucoma, with low postoperative complication rates [10,16]. GATT achieved a mean IOP reduction of 9.81 mmHg and a surgical success rate of 85%, according to a recent meta-analysis [17]. However, MIGS procedures are generally recommended for mild-to-moderate glaucoma, and there is conflicting evidence in the literature regarding the efficacy of GATT in advanced glaucoma. Grover et al. noted that the surgical failure risk after GATT increases in eyes with an MD greater than −15 [10]. However, Rahmatnejad et al. found that visual field MD scores were not statistically significant between the success and failure groups [11]. Dar et al. also did not find any correlation between MD value and surgical failure [8]. In our study, although the mean MD value was −19.79, no significant effect on surgical failure was observed. However, as we only analyzed advanced glaucoma patients, we could not make a comparison with mild and moderate MD values. On the other hand, it should be kept in mind that visual field analysis and MD values may not be reliable indicators in advanced glaucoma.

Aktaş et al. reported successful results of MIGS procedures in advanced glaucoma patients. According to their data, the success rate of GATT surgery in advanced glaucoma patients is quite high, in contrast to the low success rates reported in the literature [15].

Ahuja et al. reported that 28% of patients with advanced glaucoma required additional surgical intervention after trabectome, compared with only 10% of patients with mild-to-moderate glaucoma who did [18]. However, trabectome is not a circumferential angle surgery like GATT. This may be one of the reasons for its higher failure rate [19]. As seen in our study, the low progression rate in 360-degree GATT supports the efficacy of circumferential surgery. In another study, incomplete trabeculotomy to 360 degrees was associated with surgical failure [20]. 

The results of our study indicate that GATT surgery is significantly effective in controlling IOP and halting glaucoma progression in patients with advanced glaucoma. After surgery, there was a 40.36% reduction in IOP, which was maintained at 42.12% at final follow-up (*p* < 0.001). In addition, the number of glaucoma medications decreased by 66.23% from 3.5 preoperatively to 1.18 postoperatively, indicating that GATT was also effective in reducing the need for medication. These results are consistent with the study by Grover et al., who observed an IOP reduction of 37.3% in patients with primary open angle glaucoma (POAG) over a 24-month follow-up period [10]. Wan et al. found a 24-month success rate of 68.48% in advanced POAG, but they included drug-free status as a criterion for complete success. Their success rate with medication was 84.81%, which is compatible with our results [13]. In the study by Rahmatnejad et al., the overall success rate was 63% after a mean follow-up of 12 months. The reason for this lower success rate might be related to some patients being of African-American race. African Americans are known to have a poorer response than other races to many glaucoma surgeries, including GATT [11].

The data obtained in our study showed that factors such as age, preoperative IOP level, and postoperative IOP peak did not have a significant effect on surgical success. A study by Wang et al. in juvenile open-angle glaucoma (JOAG) patients reported that variables such as MD severity, previous glaucoma surgery, age, preoperative IOP, and medical treatment had no effect on surgical success; however, postoperative IOP peak was associated with failure [7].

In the literature, GATT has been reported to have different success rates in primary open-angle glaucoma (POAG) and secondary open-angle glaucoma (SOAG). Studies by Grover and Rahmatnejad demonstrated that greater IOP reduction was achieved in SOAG patients [10,11]. The goal IOP range for patients with PEXG must be established, and because their IOP can spike suddenly, they may require closer monitoring than individuals with POAG. [21]. Cubuk et al. reported that the IOP-lowering effect of GATT was higher in PEXG eyes (46.8% vs. 32.1%) compared to POAG patients, and the success rate reached 64.3% in the PEXG group, while it remained at 25% in the POAG group. The authors stated that GATT can provide high efficacy in eyes with PEXG due to the accumulation of pseudoexfoliative material and pigments in the proximal trabecular meshwork [22]. According to our results, there was no significant difference in surgical success between primary and secondary open-angle glaucoma patients in our long-term follow-up. However, it is noteworthy that the majority of our patients had PEXG, and this may have affected the results.

In our study, the surgical success rate of GATT in advanced glaucoma patients was 81.2% in the early period, which is consistent with the overall success rate after GATT reported in the meta-analysis by Guo et al. [17]. Although the success rate dropped to 76.5% after two years of follow-up, our study provides evidence that GATT is a safe option in advanced glaucoma patients. The decrease in the success rate may be due to the recanalization of physiological outflow pathways over time or the interruption of drop use in patients who need to continue medical treatment.

### 4.2. Clinical Implications: GATT vs. Trabeculectomy

Trabeculectomy remains the gold standard for the surgical management of advanced glaucoma, particularly in cases requiring substantial intraocular pressure (IOP) reduction. However, it carries a serious risk of complications, including endophthalmitis, hypotony, cataract formation, choroidal detachment, bleb-related complications, and unexplained central vision loss (wipe-out) [23]. Recent studies have compared the efficacy and safety profiles of GATT and trabeculectomy. Direct numerical comparisons between GATT and trabeculectomy demonstrate key differences in success rates and complication risks. The Tube vs. Trabeculectomy Study (TVT) reported a trabeculectomy failure rate of 47% at 5 years, significantly higher than the long-term success rate of 76.5% observed in our study. Additionally, trabeculectomy success rates range from 50 to 70% over five years, with complications such as the wipe-out phenomenon particularly concerning in advanced-stage patients [24,25].

A pilot randomized controlled trial by Panigrahi et al. involving patients with advanced pigmentary glaucoma reported that both GATT and trabeculectomy significantly reduced IOP and the number of glaucoma medications over a 12-month follow-up period. The absolute success rates were comparable between the two procedures, with GATT achieving 60% and 50% success by two different criteria, while trabeculectomy achieved 67.7% and 58.3%, respectively. Notably, two eyes in the trabeculectomy group developed hypotony, whereas no sight-threatening complications were observed in the GATT group [26]. In a study by Cakir et al. (2024), the efficacy and safety of GATT and trabeculectomy were compared in patients with POAG and PEXG. The results indicated that both procedures were effective in lowering IOP and reducing the number of antiglaucomatous medications [27]. Notably, GATT demonstrated a favorable safety profile with fewer postoperative complications compared to trabeculectomy [28].

Early postoperative IOP spikes, which may remain undiagnosed, could potentially cause further damage to the already compromised optic nerve in end-stage glaucoma [29]. Although postoperative IOP peak after trabeculectomy is not as common as GATT surgery, sudden IOP reductions due to excessive filtration may also cause the wipe-out phenomenon. On the other hand, given that the success of trabeculectomy is bleb-dependent, it is known that bleb-related complications decrease the long-term surgical success, even if an effective IOP reduction is achieved.

Mechanistically, trabeculectomy bypasses the conventional outflow pathway by creating a new filtration route through the sclera, which can lead to lower IOP levels but also introduces risks associated with bleb formation. GATT, on the other hand, enhances the trabecular outflow system by removing resistance at the inner wall of Schlemm’s canal and the juxtacanalicular trabecular meshwork, aiming to lower IOP to episcleral venous pressure levels. However, its efficacy may be limited in cases where the distal outflow system is compromised due to factors such as aging or structural obstructions [30].

An important reason for not preferring GATT surgery in advanced glaucoma is the belief that it may not be sufficiently effective. The trabecular outflow pathway, known as the traditional outflow pathway, is a structure that drains approximately 80–90% of the aqueous humor. The main resistance factors in this pathway are the inner wall of Schlemm’s canal and the juxtacanalicular trabecular meshwork. The removal of this resistant area by ab interno trabeculotomy provides effective IOP reduction. However, the distal outflow pathway, which accounts for 50% of the resistance in the traditional pathway, cannot be directly addressed during the procedure. In an eye with a healthy distal drainage pathway, 360° ab interno trabeculotomy is expected to lower IOP to the level of episcleral venous pressure. However, this goal might be difficult to achieve in eyes with a compromised distal drainage pathway due to factors such as aging, stasis in Schlemm’s canal, and obstruction of the collector channel ostia by the trabecular meshwork in the setting of high IOP [30,31].

### 4.3. Complications and Safety Profile

The low cost and high safety profile of GATT surgery have made it an increasingly popular MIGS technique, especially in developing countries [30]. Given the absence of complications such as blebitis, leakage, hypotony, and bleb-related endophthalmitis, GATT may be considered a safer alternative, especially in patients at high risk of complications [31].

In our study, IOP peak was observed in only two patients, and in both cases, it resolved spontaneously within the first postoperative week. No prolonged IOP peak was observed, and this parameter was not associated with surgical failure. In the literature, the incidence of IOP peak after GATT varies from 24.7% to 74%. It has been suggested that IOP peak usually occur as a result of OVD or blood cells obstructing the outflow pathway and that insufficient reserve in the post-trabecular outflow system may lead to this condition [32,33]. In the study by Rahmatnejad et al., IOP peak was observed in 44% of unsuccessful cases, while this rate was 13% in successful cases [11]. In addition, Chen et al. reported that IOP peak may increase the risk of surgical failure by 1.74 times [20]. However, some studies have reported that IOP peak is not directly related to surgical failure [8]. Salimi et al. reported that younger age was a determinant of surgical success, suggesting that trabecular occlusion in younger patients may be localized in the proximal part of the trabecular outflow pathway. This might be associated with a shorter duration of glaucoma medication and therefore less ocular surface problems in younger patients [34]. Therefore, none of the parameters analyzed alone provide sufficient evidence and should be supported by further studies.

The most common complication, consistent with the literature, was hyphema, which occurred in 34.1% of our patients. It usually resolved spontaneously and only 4.5% required anterior chamber washout. Considering that the incidence of hyphema after GATT ranges from 36% to 91.3% in the literature, with an average reported rate of 56.6% [22,35], the rate of hyphema in our study is at the lower end of this range. In the study by Aktas et al., hyphema was the most common complication after GATT (28.3%), and in the study by Rahmatnejad, it was reported that although this condition is frequent, it usually resolves spontaneously by the end of the first postoperative month [11,14]. Additionally, in patients who underwent hemi-GATT, the incidence of hyphema was found to be lower compared to 360° GATT, while maintaining a similar rate of IOP reduction. Therefore, hemi-GATT may be considered an option for patients with an active lifestyle [36].

In our study, the progression rate was higher in patients with postoperative complications. This finding may suggest that the development of complications may be a potential risk factor for glaucoma progression. However, the fact that there was no difference in terms of gender, age, GATT extent (180° or 360°), and ocular examination findings is intriguing. To understand the relationship between complication development and progression, detailed analyses of complication types should be performed in studies with larger samples.

The long-term preservation of visual acuity and peripapillary vessel density (VD) in our patients supports the safety of the surgery. Recent studies have explored the relationship between VD and glaucoma progression, particularly in the context of surgical interventions. For instance, a study found that alterations in the foveal avascular zone area correlated with IOP reduction post-surgery, suggesting that OCTA metrics can serve as valuable indicators of surgical outcomes and disease progression [37]. Yoon et al. (2021) examined changes in peripapillary and macular VD after trabeculectomy in patients with primary open-angle glaucoma. They found that a greater reduction in peripapillary VD was significantly associated with visual field deterioration post-surgery, suggesting that monitoring peripapillary VD could be crucial for assessing surgical outcomes [38].

Wipe-out was observed in only one patient, who notably had degenerative myopia. During the post-GATT follow-up, the patients’ OCTA VD values stayed constant; however, the VD of the degenerative myopic patient who suffered from wipe-out dropped. This finding suggests that, although GATT surgery is generally safe, rare but serious complications like wipe-out may occur in patients with advanced glaucoma and additional ocular pathologies. With this finding, we can say that OCTA can be used for this purpose in advanced glaucoma, which can be the subject of another study. Similarly, Özmen et al. reported a case of wipe-out following GATT in an advanced glaucoma patient [39]. Among posterior segment complications, cystoid macular edema, choroidal effusion, hypotonic maculopathy, and vitreous hemorrhage were observed at very low rates in our study, and all resolved spontaneously within one month. The rapid resolution of these rare GATT-associated complications, as noted in the literature, supports the high safety profile of this procedure [10].

### 4.4. Limitations and Future Directions

The main limitations of our study include its retrospective nature, small sample size, and the inability to perform a statistically meaningful subgroup analysis due to the limited number of cases. However, the significance of our study lies in its extended follow-up period of two years, providing both early and long-term analysis results, and its exclusive focus on patients with advanced-stage glaucoma. Moreover, this study offers valuable real-world data on the safety and efficacy of GATT in patients with advanced glaucoma. Given these findings, further prospective, multicenter trials with long-term follow-up are necessary to confirm the durability of GATT outcomes. Future research should focus on identifying predictors of surgical success, refining patient selection criteria, and directly comparing GATT with other surgical techniques in advanced glaucoma. These efforts will help establish clearer guidelines for the role of GATT in glaucoma management.

## 5. Conclusions

Considering all these results, we believe that GATT surgery may be worthwhile in patients with advanced glaucoma due to its low rates of serious complications and high success rates. The circumferential nature of GATT surgery is one of the main reasons why it could be used in moderate to advanced cases, as it provides a more comprehensive treatment of the angle compared to other MIGS techniques. However, further studies are needed to determine who may benefit most from GATT surgery. The high safety profile of GATT suggests that it should be considered before trabeculectomy in this group of patients at high risk of complications. This study confirms that GATT is safe and effective in patients with advanced glaucoma as long as careful attention is paid to surgical steps and regular follow-up frequency is ensured after surgery. Minimizing anterior chamber and IOP fluctuation during and after surgery is an important factor in reducing the risk of wipe-out in advanced glaucoma.

## Figures and Tables

**Table 1:** Baseline demographics and clinical characteristics of study participants.

Parameter	Value	Percentage (%)	Number of Patients
Total Number of Patients			44
Gender			
-Male		84.1	37
-Female		15.9	7
Laterality			
-Right Eye		51.1	23
-Left Eye		48.9	21
Lens Status			
-Phakic		79.5	35
-Pseudophakic		20.5	9
Age (Mean ± SD) Range: 17–83 years	60.29 ± 15.15		
Central Corneal Thickness	523.40 ± 34.49 µm		
Follow-up Duration	2.69 ± 0.60 years		
Patients with Additional Ocular Pathology		11.4	5
Patients Without Any Progression		66.0	29
Glaucoma Etiologies			
-Pseudoexfoliation (PEX) Glaucoma		61.4	27
-Primary Open-Angle Glaucoma (POAG)		20.5	9
-Pigment Dispersion Syndrome (PDS)		6.8	3
-Juvenile Glaucoma		6.8	3
-Secondary to Fuchs’ Uveitis		2.3	1
-Post-traumatic Angle Recession		2.3	1
GATT Procedure Extent			
-360 degrees		79.5	35
-180 degrees		20.5	9
Complications			
-No complication		56.8	25
-Hyphema (no intervention required)		34.1	15
-Hyphema (anterior chamber washout)		4.5	2
-Permanent vision loss		4.5	2
-Urrets-Zavalia syndrome		2.3	1
-Hypotonic maculopathy with choroidal folds		2.3	1

**Table 2:** Key changes in visual and structural parameters before and after GATT surgery.

Parameter	Preoperative (Mean ± SD)	Postoperative 3rd Month (Mean ± SD)	Final (Mean ± SD)	*p*-Value (Pre-3rd)	*p*-Value (Pre-Final)
Visual Acuity (LogMAR)	0.61 ± 0.36	0.41 ± 0.33	0.59 ± 0.35	0.011	1.00
Intraocular Pressure (IOP) (mmHg)	26.9 ± 10.4	13.8 ± 8.3	13.9 ± 4.0	<0.001	<0.001
Mean Deviation (MD) (dB)	−19.79 ± 9.11	−19.03 ± 9.70	−19.06 ± 9.27	1.000	1.000
Visual Field Index (VFI)	52.25 ± 3.51	50.32 ± 2.11	51.28 ± 2.90	1.000	1.000
Mean RNFL (µm)	51.97 ± 13.09	52.76 ± 13.51	49.94 ± 12.37	1.000	1.000

**Table 3:** Factors associated with postoperative complications following GATT surgery.

Variable	No Complications (*n* = 19)	Complications (*n* = 25)	Test Statistic	Test Used	*p*-Value
Gender	Female: 3 (15.8%) Male: 16 (84.2%)	Female: 4 (16.0%) Male: 21 (84.0%)	χ^2^ = 0.0	Chi-Square Test	1.000
GATT Procedure	360 degrees: 16 (84.2%) 180 degrees: 3 (15.8%)	360 degrees: 19 (76.0%) 180 degrees: 6 (24.0%)	χ^2^ = 0.085	Chi-Square Test	0.093
Lens Status	Phakic: 15 (78.9%) Pseudophakic: 4 (21.1%)	Phakic: 20 (80.0%) Pseudophakic: 5 (20.0%)	χ^2^ = 0.0	Chi-Square Test	1.000
Postoperative Progression	No Progression: 16 (84.2%) Progression: 3 (15.8%)	No Progression: 13 (48.1%) Progression: 14 (51.9%)	χ^2^ = 4.77	Chi-Square Test	0.029
Age (years) (median, min-max)	73 (55–83)	65 (17–78)	U = 206.5	Mann–Whitney U Test	0.274
Central Corneal Thickness (CCT) (µm) (median, min-max)	533 (486–581)	510 (477–538)	U = 205	Mann–Whitney U Test	0.397
Preoperative Visual Acuity (logMAR) (median, min-max)	0.22 (0–0.3)	0.05 (0–0.4)	U = 203.5	Mann–Whitney U Test	0.410
Preoperative IOP (mmHg) (median, min-max)	30 (18–43)	26 (12–40)	U = 207	Mann–Whitney U Test	0.679
Preoperative MD (dB) (median, IQR)	−27.38; IQR: 10.63	−18.92; IQR: 18.11	U = 206.5	Mann–Whitney U Test	0.421
Preoperative PSD (dB) (median, IQR)	6.44; IQR: 4.46	8.45; IQR: 4.45	U = 205.5	Mann–Whitney U Test	0.429
Preoperative RNFL (µm) (median, min-max)	50 (36–67)	48 (38–51)	U = 203	Mann–Whitney U Test	0.432
ppVD_total_preop (%) (median, min-max)	34(26–48)	30 (27–40)	U = 204	Mann–Whitney U Test	0.431

Categorical data are presented as frequencies and percentages. Continuous data are summarized using mean ranks due to the use of the Mann–Whitney U test. Chi-Square Test: The Chi-Square test was used to assess the associations between categorical variables (e.g., gender, GATT procedure extent, lens status, postoperative progression) and the presence or absence of complications. Mann–Whitney U Test: The Mann–Whitney U test, a non-parametric alternative to the independent-samples *t*-test, was employed to compare continuous variables (e.g., age, central corneal thickness, preoperative measurements) between the two groups. The Mann–Whitney U test was chosen because the data did not meet the assumptions of normality required for parametric tests, as assessed by the Shapiro–Wilk test (not shown) or due to small sample sizes. A *p*-value less than 0.05 was considered statistically significant. VA: visual acuity; IOP: intraocular pressure; RNFL: retinal nerve fiber thickness; MD: mean deviation; PSD: pattern standard deviation; ppVD: total peripapillary vessel density; IQR: interqurtile range.

**Table 4:** Risk factors for progression after GATT surgery.

Categorical Variables				
Variable	No Progression (*n* = 29)	Progression (*n* = 15)	Test Statistic	*p*-Value
Gender				
-Female	5 (17.2%)	2(13.3%)	χ^2^ = 0.0	1.000
-Male	24 (82.8%)	13 (86.7%)		
GATT Procedure				
-360 degrees	27 (93.1%)	8 (53.3%)	χ^2^ = 7.32	0.006
-180 degrees	2 (6.9%)	7 (46.7%)		
Lens Status				
-Phakic	25 (86.2%)	10 (66.7%)	χ^2^ = 1.27	0.259
-Pseudophakic	4 (13.8%)	5 (33.3%)		
Continuous Variables				
Variables	Median (min–max)	Median (min–max)	Mann–Whitney U	*p*-value
Age (years) (median, min–max)	60 (17–83)	64 (42–78)	102.0	0.899
Central Corneal Thickness (CCT) (µm) (median, min–max)	512 (477–581)	524 (510–538)	15.0	0.602
Preoperative VA (logMAR) (median, min–max)	0.22 (0.0–1)	0.15 (0.0–1)	108.0	0.353
Preoperative IOP (mmHg) (median, min–max)	27 (11–60)	23 (12–40)	118.0	0.443
Preoperative MD (dB) (median, IQR)	−29.2; IQR: 5.95	−18.40; IQR: 17.11	50.0	0.250
Preoperative PSD (dB) (median, IQR)	6.4; IQR: 3.33	7.9; IQR: 6.69	52.0	0.304
Preoperative RNFL (µm) (median, min–max)	48 (26–68)	50 (35–87)	134.0	0.909
ppVD_total_preop (%) (median, min–max)	35 (26–46)	32 (27–48)	10.0	0.117

Categorical data are presented as frequencies and percentages. Continuous data are summarized using mean ranks due to the use of the Mann–Whitney U test. Chi-Square Test: The Chi-Square test was used to assess the associations between categorical variables (e.g., gender, GATT procedure extent, lens status, postoperative progression) and the presence or absence of complications. Mann–Whitney U Test: The Mann–Whitney U test, a non-parametric alternative to the independent-samples *t*-test, was employed to compare continuous variables (e.g., age, central corneal thickness, preoperative measurements) between the two groups. The Mann–Whitney U test was chosen because the data did not meet the assumptions of normality required for parametric tests, as assessed by the Shapiro–Wilk test (not shown) or due to small sample sizes. A *p*-value less than 0.05 was considered statistically significant. VA: visual acuity; IOP: intraocular pressure; RNFL: retinal nerve fiber thickness; MD: mean deviation; PSD: pattern standard deviation; ppVD: total peripapillary vessel density; IQR: interqurtile range.

**Table 5:** Comparison of IOP reduction outcomes in key GATT studies.

Study	Glaucoma Type	N	Mean Preop IOP (mmHg)	IOP at 3 M (mmHg)	% Reduction at 3M	IOP at 24 M (mmHg)	% Reduction at 24 M	Success Rate (24 M)
Current Study	Advanced OAG	44	26.9 ± 10.4	13.8 ± 8.3	40.36%	13.9 ± 4.0	42.12%	76.5%
Grover et al. [10] (2018)	POAG	157	23.2 ± 7.1	Not Reported	Not Reported	14.5 ± 3.6	37.3%	75.3%
Wan et al. [13] (2022)	POAG + PEXG	66	25.4 ± 5.1	Not Reported	Not Reported	14.3 ± 4.7	43.7%	84.8% (with meds)
Wang et al. [7] (2023)	OAG w/failed surgery	49	28.3 ± 5.2	16.0 ± 4.7	43.5%	16.7 ± 5.1	41.1%	71.4%
Dar et al. [8] (2023)	Advanced OAG	41	27.1 ± 6.3	15.6 ± 3.8	42.4%	15.2 ± 4.2	44.1%	72.0%
Rahmatnejad et al. [11] (2017)	POAG + PEXG	85	24.5 ± 6.2	15.2 ± 4.9	38.0%	Not Reported	Not Reported	63.0% (12M)
Sharkawi et al. [16] (2021)	PEXG	60	27.8 ± 5.3	16.2 ± 5.1	41.7%	16.5 ± 5.4	40.6%	78.2%
Aktaş et al. [15] (2018)	Advanced OAG	35	27.3 ± 5.9	15.1 ± 4.6	44.7%	15.5 ± 4.1	43.2%	79.3%
Aktaş et al. [14] (2019)	Moderate–Advanced OAG	42	26.7 ± 6.4	14.9 ± 4.8	44.2%	15.1 ± 4.5	43.0%	77.5%

## Data Availability

The data presented in this study are available on request from the corresponding author. (the data are not publicly available due to privacy or ethical restrictions).
